# Family child care home providers’ self-reported nutrition and physical activity practices, self-efficacy, barriers and knowledge: baseline findings from happy healthy homes

**DOI:** 10.1017/S1368980022000337

**Published:** 2022-08

**Authors:** Susan B Sisson, Erin Eckart, Bethany D Williams, Sarah M Patel, Chelsea L Kracht, Holly A Davis, Dianne S Ward, Deana Hildebrand, Julie A Stoner, Emily Stinner, Kelly E Kerr, Alicia Salvatore

**Affiliations:** 1 Department of Nutrition Sciences, University of Oklahoma Health Sciences Center, 1200 N Stonewall Ave, AHB 3057, Oklahoma City, OK 73117-1215, USA; 2 Department of Biostatics and Epidemiology, University of Oklahoma Health Sciences Center, Oklahoma City, OK, USA; 3 Department of Nutrition and Exercise Physiology, Elson S. Floyd College of Medicine, Washington State University Health Sciences Spokane, Spokane, WA, USA; 4 Pennington Biomedical Research Center, Baton Rouge, LA, USA; 5 University of North Carolina Chapel Hill, Chapel Hill, NC, USA; 6 Oklahoma State University, Stillwater, OK, USA; 7 Value Institute, ChristianaCare, Neward, DE, USA

**Keywords:** Early care and education, Preschool, Diet, Nutrition, Movement, Physical activity, Food programme

## Abstract

**Objective:**

Describe nutrition and physical activity practices, nutrition self-efficacy and barriers and food programme knowledge within Family Child Care Homes (FCCH) and differences by staffing.

**Design:**

Baseline, cross-sectional analyses of the Happy Healthy Homes randomised trial (NCT03560050).

**Setting:**

FCCH in Oklahoma, USA.

**Participants:**

FCCH providers (*n* 49, 100 % women, 30·6 % Non-Hispanic Black, 2·0 % Hispanic, 4·1 % American Indian/Alaska Native, 51·0 % Non-Hispanic white, 44·2 ± 14·2 years of age. 53·1 % had additional staff) self-reported nutrition and physical activity practices and policies, nutrition self-efficacy and barriers and food programme knowledge. Differences between providers with and without additional staff were adjusted for multiple comparisons (*P* < 0·01).

**Results:**

The prevalence of meeting all nutrition and physical activity best practices ranged from 0·0–43·8 % to 4·1–16·7 %, respectively. Average nutrition and physical activity scores were 3·2 ± 0·3 and 3·0 ± 0·5 (max 4·0), respectively. Sum nutrition and physical activity scores were 137·5 ± 12·6 (max 172·0) and 48·4 ± 7·5 (max 64·0), respectively. Providers reported high nutrition self-efficacy and few barriers. The majority of providers (73·9–84·7 %) felt that they could meet food programme best practices; however, knowledge of food programme best practices was lower than anticipated (median 63–67 % accuracy). More providers with additional staff had higher self-efficacy in family-style meal service than did those who did not (*P* = 0·006).

**Conclusions:**

Providers had high self-efficacy in meeting nutrition best practices and reported few barriers. While providers were successfully meeting some individual best practices, few met all. Few differences were observed between FCCH providers with and without additional staff. FCCH providers need additional nutrition training on implementation of best practices.

Unhealthy childhood behaviours, including intake of nutrient-poor foods and insufficient physical activity, may contribute to excess weight gain, suboptimal growth and development and chronic disease^(1–3)^. It is important to understand the influence of key environments in which young children spend substantial time and engage with significant caregivers on nutrition and physical activity. Childcare providers are in strategic positions, as many children spend substantial amounts of time in their care, and they may have a strong influence on these obesogenic behaviours^(2,4)^. Family Child Care Homes (FCCH) are a unique childcare context compared with centre-based care, since FCCH serve a varied age range of children in a single space; are primarily conducted in a home-based setting; typically have a single owner/caregiver, though some may have additional staff and do not have food service staff^(5)^. Moreover, children who receive care at FCCH may be at increased risk for overweight/obesity^(6)^, and FCCH are frequently used by low-income families due to flexible hours and lower costs^(7)^.

To support childcare providers serving low-income children, the United States food programme reimburses qualifying providers for food costs and provides best practice recommendations. Approximately 78 % of the FCCH in the USA participate in the food programme^(8,9)^. Food programme participation is associated with children’s enhanced nutrition^(10)^ and best practices^(10,11)^. However, there are variations in the fidelity with which the food programme is implemented in FCCH and centre-based programmes^(10)^. Implementation variations may be the result of provider training^(11)^, nutrition and food programme knowledge^(12)^ or self-efficacy.

FCCH are meeting some desired best practices, such as serving fruits and vegetables daily^(11,14,15)^. Jiang *et al.*^(13)^ report that some FCCH attitudes toward meal environments and foods served align with the food programme requirements and aspirational best practices, but there are opportunities to strengthen alignment and more research is needed. Previous research demonstrates that provider feeding and mealtime practices can positively or negatively influence children’s dietary intake and willingness to try foods^(16–19)^. Higher quality FCCH-level nutrition policies are related to children’s healthier dietary intake^(20)^ and underscore the importance of this child care setting. However, few studies describe FCCH provider food programme knowledge and nutrition and feeding self-efficacy and barriers that may influence the foods served and mealtime best practices.

Along with providing adequate nutrition, providers must facilitate physical activity, and provider physical activity, attitudes and beliefs can influence children’s movement^(21,22)^. Higher quality FCCH-level physical activity policies are related to higher levels of daily physical activity^(23)^. However, there is substantial room for improving health practices, especially regarding physical activity^(11,15,24)^, which are less consistently emphasised in state licensure policy^(25)^. Unlike nutrition, there is no financial incentive for adequate physical activity in childcare. Currently, evidence is inconclusive as to the influence of the FCCH physical activity environment^(26)^. Other research in centre-based programmes has found that access to play equipment, outdoor spaces and provider engagement in child physical activity is generally associated with higher levels of physical activity in young children^(26)^. A recent review of state licensing standards reported that 27 % of the Caring for our Children recommendations for physical activity, safety and outdoor play were met by FCCH^(25)^. However, FCCH had few written policies regarding nutrition and physical activity compared with centre-based programmes^(15)^. Limited policy and training opportunities may be a potential explanation for low adherence^(11,15)^. Therefore, FCCH play a critical role in the development of children in their care as the sole nutrition and physical activity provider during hours in care.

FCCH with multiple staff members may be at an advantage of meeting nutrition and physical activity practices and having higher self-efficacy and lower barriers due to the additional support within this care setting. The additional staff may provide additional supervision and attention to children, while others prepare meals, which may deter against sedentary activities (e.g. screen-time) and promote physical activity. On the other hand, more staff could deter these best practices with additional staff members to model and reinforce unhealthy behaviours. This critical component of administering and demonstrating healthy practices is unique to FCCH with implications for future training and administration within this context.

Taken together, the first aim of the current study is to characterise FCCH nutrition and physical activity practices, policies, nutrition self-efficacy and barriers and food programme knowledge in a sample of FCCH providers in Oklahoma. The second aim of the current study explores these differences by additional staff, with the hypothesis that FCCH providers with additional staff may be more likely to meet nutrition and physical activity best practices than are FCCH providers without additional staff.

## Materials and methods

### Study design

The current study examined baseline measures of Happy Healthy Homes, a randomised attention-matched controlled trial of FCCH providers, described elsewhere^(27)^. Providers were recruited through food programme sponsoring organisations and direct phone calls to FCCH. Inclusion criteria were participation in the food programme, serving at least one child who was 2-to-5 years old, being located within 60 miles of the metro area, and planning to remain in business for at least 12 months. Recruitment goals were based on the necessary power for the intervention effect^(27)^. A financial incentive of $30 was provided for baseline. Data were collected between October 2017 and November 2018.

### Measures

Providers completed online or paper surveys to ascertain FCCH provider demographic characteristics, nutrition and physical activity practices and policies, nutrition self-efficacy and barriers and food programme knowledge. Providers shared time spent in FCCH food preparation (open-ended), types of food preparation methods (checklist), timing of meal preparation (checklist) and what children do during meal preparation (open-ended). While it is not possible to fully remove social desirability bias, providers were encouraged to provide honest answers. Participants were reminded by a trained research assistant that their responses would remain confidential and that there were ‘no right or wrong answers’ to surveys.

### Nutrition and physical activity best practices

Nutrition and physical activity practices and policies were reported using the validated FCCH Nutrition and Physical Activity Self-Assessment^(28)^. The nutrition and physical activity components include forty-three and sixteen questions, respectively. Each item has four response options, each with a possible best practice. The response items are scored as one through four, with four being the best practice for the individual item. Example questions and response items follow. An example nutrition item is, ‘My program offers fruit:’ with response options ‘≤3 times/week’ (1 point), ‘4 times/week’ (2 points), ‘once/d’ (3 points) and ‘≥2 times/d’ (4 points/best practice). An example of physical activity item is ‘The amount of time I provide for children’s indoor and outdoor physical activity each day is’ with response options ‘<60 min/d’ (1 point), ‘60–74 min/d’ (2 points), ‘75–89 min/d’ (3 points) and ‘≥90 min/d’ (4 points/best practice). The prevalence of individual best practices and best practices within survey sections were calculated. There are seven nutrition sections and five physical activity sections. Scores for the overall instrument and scores within each section were averaged and summed.

### Nutrition self-efficacy and barriers

Provider nutrition self-efficacy (eighteen questions) and barriers (twenty questions) were evaluated^(29)^. An example item assessing self-efficacy is “How sure are you that you can serve the children vegetables 2 or more time a day?” Response options included “not at all sure,” “a little sure,” “sure” and very sure.” An example item assessing barriers is “You have enough time to prepare healthy food as often as you would like?” Response options included “agree a lot,” “agree a little,” “neither agree or disagree,” “disagree a little” and “disagree a lot.” Likert response options were given numerical values and summed across respective sections. Appropriate items were reverse scored. The possible range of scores for self-efficacy was 0–18, with a higher score indicating higher self-efficacy (Cronbach’s *α* = 0·84). “Sure” and “very sure” responses were collapsed for reporting self-efficacy. The possible range of scores for barriers was 20–60, with a lower score indicating fewer barriers (Cronbach’s *α* = 0·70). “Agree a little” and “agree a lot” were collapsed for reporting.

### Food programme knowledge

Fourteen questions evaluated the provider’s knowledge specific to the food programme requirements and best practices^(30)^. Example knowledge questions are, ‘Avoiding any fruit juice is a best practice (yes/no);’ ‘Family-style meal service requires that the full portion be on the child’s plate’ (true/false). The sum of correct answers and overall percent of accuracy were calculated. The possible range of scores was 0–13, with a higher score indicating higher food programme knowledge.

### Data analysis

A total of fifty-one providers were recruited. Two did not provide complete study responses and were removed from analyses, yielding an analytical sample size of forty-nine FCCH providers. For aim one, central tendencies were calculated, and normality was assessed with a Shapiro–Wilk test for variables (food programme knowledge, nutrition self-efficacy and barriers, nutrition and physical activity practices and policies) due to non-normal distribution of variables. Free-response options for what children do while the provider prepares meals were examined for content and categorised into the following categories: free play, watch TV, directed learning activity, exercise and help get ready for the meal (set table, wash hands, etc.). For aim two, a *χ*^2^ or Fisher’s exact analysis (categorical data) or an independent *t*-test (parametric data) or Wilcoxon rank sum test (non-parametric data) was used to evaluate differences between providers with and without additional staff. The *α* level was examined at <0·01 to reduce error from multiple comparisons. All analyses were conducted using SAS 9.4.

## Results

### Demographic characteristics

**Fig. 1:**
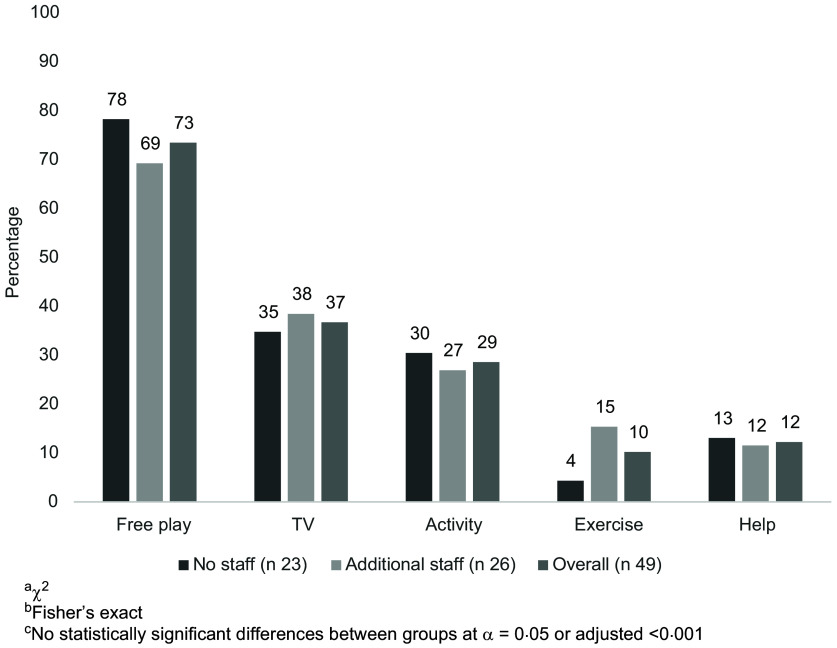
Family child care home-reported children’s activity during meal preparation (*n* 49)

All participants were women. Participants were 51·0 % Non-Hispanic White, 30·6 % Non-Hispanic Black, 2·0 % Hispanic, 4·1 % America Indian/Alaska Native and 44·2 ± 14·2 years of age (Table 1). Slightly over half (*n* 26, 53·1 %) of providers had additional staff. Overall, providers spent 2·0 h/d in food preparation, predominantly the night before (46·9 %), in the morning before (55·1 %) and after the children arrived (71·4 %). There were no demographic differences between providers with and without additional staff, with one exception (Table 1). Providers with additional staff cared for more children than did those without (median of 12 *v*. 7, *P* = 0·0012), which was anticipated as larger programmes require more staff to maintain licensing ratios. Providers utilised a variety of food preparation methods, besides deep-frying, and many believed that the food programme helped them provide healthier meals for children (91·8 %). As shown in Fig. 1, the most common activity in which children participated during meal preparation was free play, followed by watching TV, directed learning activities, helping get ready for the meal and engaging in exercise.

**Table 1:** Demographic characteristics of family child care home (FCCH) providers with and without additional staff in and around Oklahoma City participating in happy healthy homes baseline measures fall 2017–fall 2018 (*n* 49)

	Providers with other staff (*n* 26)		Providers without other staff (*n* 23)		Overall (*n* 49)	
	Mean	sd	Mean	sd	Mean	sd
Provider characteristics						
Age (years)§	46·8	14·7	40·9	13·1	44·2	14·2
Number of adults living in the household‖	2·0	2·0, 2·0	2·0	2·0, 2·0	2·0	2·0, 2·0
Number of children living in the household‖	0	0, 2·0	2·0	0, 3·0	2·0	0, 3·0
Race/Ethnicity¶	*n*	%	*n*	%	*n*	%
Hispanic White	0	0	1	4·4	1	2·0
Non-Hispanic White	15	57·7	10	43·5	25	51·0
Non-Hispanic Black	7	26·9	8	34·5	15	30·6
American Indian or Alaska Native	2	7·7	0	0	2	4·1
Other	1	3·9	3	13·0	4	8·2
Do not wish to provide	1	3·9	1	4·4	2	4·1
Highest level of education attained¶						
High school graduate or GED	2	7·7	2	8·7	4	8·2
Some college or vocational training	15	57·7	16	69·6	31	63·3
4-year college graduate or higher	9	34·6	5	21·7	14	28·6
General health¶						
Excellent	2	7·7	1	4·4	3	6·1
Very good	11	42·3	14	60·9	25	51·0
Good	12	46·2	8	34·8	20	40·8
Fair	1	3·9	0	0	1	2·0
Degree in early child education or development††	9	34·6	6	27·3	15	31·3
Child Development Associate (CDA) credential¶	7	28·0	5	21·7	12	25·0
Household income¶						
$15 000–$34 999	4	15·4	5	21·7	9	18·4
$35 000–$49 999	3	11·5	5	21·7	8	16·3
$50 000–$99 999	10	38·5	7	30·4	17	34·7
$100 000–$199 999	5	19·2	5	21·7	10	20·4
Do not wish to provide	4	15·4	1	4·4	5	10·2
Programme Characteristics						
	Mean	sd	Mean	sd	Mean	sd
Number of supervised children 5 years and younger**,§	8·3	3·6	5·1	2·8	6·8	3·6
Number of supervised children related to provider‖	1·0	0, 2·0	2·0	1·0, 2·0	1·0	0, 2·0
Year of business‖	9·3	4·0, 17·0	6·0	1·5, 15·0	8·0	2·0, 17·0
Number of Children Supervised**,‖	12·0	9·0, 12·0	7·0	6·0, 8·0	9·0	7·0, 12·0
	*n*	%	*n*	%	*n*	%
1–5**,¶	1	3·9	3	13·0	4	8·2
6–10	10	38·5	17	73·9	27	55·1
11–15	11	42·3	3	13·0	14	28·6
16–20	2	7·7	0	0	2	4·1
21–25	2	7·7	0	0	2	4·1
Star level (AKA QRIS rating)¶						
1	10	38·5	9	39·1	19	38·8
1+	4	15·4	5	21·7	9	18·4
2	9	34·6	5	21·7	14	28·6
3	2	7·7	0	0	2	4·1
Do not know/not sure	1	3·9	4	17·4	5	10·2
Programme food and nutrition characteristics						
Average number of h/d in food preparation for FCCH†,‖	2·0	1·5, 2·5	2·0	1·0, 2·5	2·0	1·0, 2·5
Time meals are typically prepared‡	*n*	%	*n*	%	*n*	%
Morning before kids arrive	14	53·9	13	52·5	27	55·1
Morning after kids arrive	21	80·8	14	60·9	35	71·4
Night before	12	46·2	11	47·8	23	46·9
Weekend	5	19·2	7	30·4	12	24·5
Other	6	23·1	3	13·0	9	18·4
Types of food preparation methods used in the FCCH‡						
Baking/roasting in oven	26	100·0	23	100·0	49	100·0
Boiling/stewing/simmering¶	20	76·9	19	82·6	39	79·6
Deep frying	0	0	0	0	0	0
Grilling/broiling††	15	57·7	10	43·5	25	51·0
Microwaving††	17	65·4	17	73·9	34	69·4
Pan-frying/sautéing††	15	57·7	15	65·2	30	61·2
Pressure cooking††	7	26·9	4	17·4	11	22·5
Slow cooking in crock pot††	19	73·1	16	69·6	35	71·4
Steaming††	15	57·7	15	65·2	30	61·2
Other¶	4	15·4	1	4·4	5	10·2
Do you think the food programme helps you provide healthier meals¶	22	84·6	23	100·0	45	91·8
Do you consider the food programme paperwork to be excessive¶	5	19·2	5	21·7	10	20·4

† Participants could write in open response.

‡ Participants could select multiple response options.

§ *t*-test for independent means.

‖ Wilcoxon.

¶ Fisher’s exact.

†† *χ*^2^.

Continuous data are reported as means ± sd or medians (25th, 75th percentile) and analysed using *t*-test for independent means or Wilcoxon Rank Sum, as appropriate.

Statistical significance was examined at alpha < 0·05 (*) and < 0·01 (**), to account for multiple analyses.

### Nutrition and physical activity practices, nutrition self-efficacy and barriers and food programme knowledge

Table 2 shows the numerical score and prevalence of meeting aspirational best practices for each section and each individual practice. The prevalence of meeting nutrition best practices was poorest for feeding environment (no FCCH met best practices for the entire section) and highest for menu and variety (43·8 % met best practices for the entire section). The prevalence of meeting physical activity best practices was lowest for indoor play equipment (4·1 % met best practices for the entire section) and highest for daily physical activity practices (16·7 % met best practices for the entire section, Table 2). The average nutrition practices score was 3·2 ± 0·3 (max 4·0). The sum nutrition practices score was 137·5 ± 12·6 (possible range 43 minimum – 172 maximum; Table 2). The average physical activity practices score was 3·0 ± 0·5 (max 4·0). The sum physical activity practices score was 48·4 ± 7·5 (possible range 16 minimum – 64 maximum; Table 2). There were no differences between FCCH with and without additional staff.

**Table 2:** Prevalence of best practice nutrition and physical activity practices and policies of family child care home providers in and around Oklahoma City (*n* 49)

	Providers with other staff (*n* 26)		Providers without other staff (*n* 23)		Overall (*n* 49)	
	Mean	sd	Mean	sd	Mean	sd
Nutrition total sum score§ Min – Max possible score: 43 – 172	139·2	10·6	135·5	14·5	137·5	12·6
Nutrition total average score§	3·2	0·2	3·2	0·3	3·2	0·3
Foods provided	Median	IQR	Median	IQR	Median	IQR
Average foods provided subsection score‖	3·5	2·9, 3·7	3·4	3·2, 3·5	3·5	3·1, 3·6
	Count	%	Count	%	Count	%
Best Practice for all Foods Provided Questions‡	2	8·3	0	0·0	2	4·3
Offers fruit ≥2/d†	15	57·7	11	47·8	26	53·1
Offers fruit that is fresh, frozen or canned in juice every time†	15	57·7	18	78·3	33	67·4
Offers vegetables ≥2/d†	12	46·2	6	26·1	18	36·7
Offers dark green, orange, red or deep yellow vegetables ≥1/d†	9	34·6	8	34·8	17	34·7
Rarely or never offers vegetables cooked with meat fat, margarine or butter†	14	53·9	8	34·8	22	44·9
Offers fried or pre-fried potatoes ≤1/week†	17	68·0	19	82·6	36	75·0
Offers fried or pre-fried meat or fish ≤1/week†	19	76·0	18	78·3	37	77·1
Offers high-fat meats ≤1/week†	16	61·5	13	56·5	29	59·2
Offers lean or low-fat meat alternates every time served‡	6	23·1	4	17·4	10	20·4
Offers high fibre, whole grain foods ≥2/d†	10	38·5	8	34·8	18	36·7
Offers high-sugar, high-fat foods ≤1/week‡	22	84·6	20	87·0	42	85·7
Offers high-salt, high-fat snacks ≤1/week‡	19	73·1	21	91·3	40	81·6
Offer sweet or salty snacks outside of meal/snack time ≤1/week‡	21	80·8	19	82·6	40	81·6
Beverages provided	Median	IQR	Median	IQR	Median	IQR
Average beverages provided subsection score‖	3·6	3·0, 3·8	3·4	3·2, 3·8	3·6	3·2, 3·8
	Count	%	Count	%	Count	%
Best practice for all beverages provided questions†	10	45·5	6	26·1	16	35·6
Drinking water is visible and freely available indoors and outdoors†	20	76·9	17	73·9	37	75·5
Offers 4–6 oz. serving 100 % fruit juice ≤2/week†	18	78·3	15	65·2	33	71·7
Never offers sugary drinks†	21	80·8	17	73·9	38	77·6
Offers low fat or fat-free milk for children over 2 years‡	21	80·8	18	78·3	39	79·6
Never offers flavoured milk‡	20	80·0	21	91·3	41	85·4
Feeding environment	Median	IQR	Median	IQR	Median	IQR
Average feeding environment subsection score‖	2·9	2·7, 3·1	2·9	2·6, 3·1	2·9	2·6, 3·1
	Count	%	Count	%	Count	%
Best practice for all feeding environment questions	0	0·0	0	0·0	0	0·0
Children always choose and serve most/all foods themselves‡	2	8·0	0	0·0	2	4·2
TV and videos are never on during meal/snack times*,†	20	76·9	11	47·83	31	63·3
Provider always eats and drinks same food and beverages as children‡	2	7·7	3	13·0	5	10·2
Provider rarely/never eats or drinks unhealthy food or beverage in view of children†	18	69·2	18	78·3	36	73·5
Provider enthusiastically role models eating healthy foods at every meal/snack†	6	23·1	6	26·1	12	24·5
Programme has large variety of healthy lifestyle materials with new items added and rotated‡	5	19·2	4	17·4	9	18·4
Programme has few posters, books, materials that promote unhealthy food†	17	65·4	19	82·6	36	73·5
Feeding Practices	Median	IQR	Median	IQR	Median	IQR
Average Feeding Practices Subsection Score‖	3·4	3·2, 3·6	3·4	3·1, 3·7	3·4	3·2, 3·6
	Count	%	Count	%	Count	%
Best Practice for all Feeding Practices Questions‡	0	0·0	2	8·7	2	4·1
Provider always praises children for trying new or less-preferred foods†	19	73·1	15	65·2	34	69·4
Provider always asks children about fullness before removing plate ≤ half consumed†	20	76·9	16	69·6	36	73·5
Provider always asks children about hunger when second servings are requested†	7	26·9	9	39·1	16	32·7
Provider rarely or never requires children to sit at the table and clean their plate‡	24	92·3	19	82·6	43	87·8
Provider uses authoritative feeding style every meal and snack†	11	42·3	8	34·8	19	38·8
Provider rarely or never uses child’s preferred foods to encourage consumption†	16	61·5	12	52·2	28	57·1
Provider rarely or never uses food to calm or encourage children‡	24	92·3	20	86·9	44	89·8
Provider always praises and provides hands-on help to guide toddler self-feeding†	16	61·54	14	60·9	30	61·2
At ≥1/play period providers reminder children to drink water during physically active play†	3	11·5	8	34·8	11	22·5
Menus and Variety	Median	IQR	Median	IQR	Median	IQR
Average Menus and Variety Subsection Score‖	3·5	3·5, 4·0	3·5	3·5, 4·0	3·5	3·5, 4·0
	Count	%	Count	%	Count	%
Best Practice for all Menus and Variety Questions†	12	48·0	9	39·1	21	43·8
Programme menu is ≥3 weeks and changes seasonally†	15	60·0	10	43·5	25	52·1
Programme weekly menu always includes healthy foods‡	23	88·5	22	95·7	45	91·8
Nutrition Education and Professional Development	Median	IQR	Median	IQR	Median	IQR
Average Nutrition Education & Professional Development Subsection Score‖	3·1	2·8, 3·4	2·6	2·0, 3·2	3·0	2·4, 3·4
	Count	%	Count	%	Count	%
Best Practice for all Education and Professional Development Questions‡	2	7·7	2	9·52	4	8·5
Provider leads planned nutrition education ≥1/week†	8	30·8	5	21·7	13	26·5
Provider talks informally with children about healthy eating at each opportunity†	11	42·3	7	30·4	18	36·7
Provider completes professional development of child nutrition ≥2/year†	8	30·8	5	22·7	13	27·1
Provider has covered 5–6 topics in nutrition professional development*,†	16	61·5	7	30·4	23	46·9
Provider offers families information on child nutrition ≥2/year†	13	50·0	10	45·5	23	47·9
Provider offers families information 5–6 child nutrition topics†	9	34·6	6	27·3	15	31·3
Nutrition Policy	Median	IQR	Median	IQR	Median	IQR
Average Nutrition Policy Subsection Score‖	3·0	2·0, 3·0	2·0	2·0, 3·0	2·0	2·0, 3·0
	Count	%	Count	%	Count	%
Best Practice on Program Nutrition Policy Includes 6–9 Nutrition Topics‡	5	20·0	5	21·7	10	20·8
	Mean	sd	Mean	sd	Mean	sd
Physical Activity Total Sum Score Min – Max possible score: 16 – 64§	49·5	6·4	47·3	8·6	48·4	7·5
Physical Activity Total Average Score§	3·1	0·4	3·0	0·5	3·0	0·5
Time Provided for Physical Activity	Median	IQR	Median	IQR	Median	IQR
Average time provided for physical activity subsection score‖	3·0	2·7, 3·7	3·0	2·7, 3·3	3·0	3·0, 3·3
	Count	%	Count	%	Count	%
Best Practice for all Time Provided for Activity and Sedentary Behaviour Questions‡	4	15·4	2	8·7	6	12·2
Time provided for indoor and outdoor physical activity is ≥90 min/d†	15	57·7	10	43·5	25	51·0
Adult-led physical activity is ≥45 min/d†	8	30·8	7	30·4	15	30·6
Outside of nap and meal times, children are seated for ≤15 min at a time†	13	50·0	13	56·2	26	53·1
Indoor Play Environment	Median	IQR	Median	IQR	Median	IQR
Average indoor play environment subsection score‖	3·3	3·0, 3·7	3·0	2·3, 3·7	3·0	2·7, 3·7
	Count	%	Count	%	Count	%
Best Practice for all Indoor Play Equipment Questions†	1	3·9	1	4·4	2	4·1
Programme has ≥4 types of portable play equipment†	19	73·1	13	56·5	32	65·3
During indoor free play time, a few portable play toys are always available†	9	34·6	9	39·1	18	36·7
Programme has large variety of posters, books and materials that promote physical activity‡	7	26·9	2	8·7	9	18·4
Daily Physical Activity Practices	Median	IQR	Median	IQR	Median	IQR
Average daily physical activity practices subsection score‖	3·7	3·3, 3·7	3·3	3·0, 3·7	3·3	3·0, 3·7
	Count	%	Count	%	Count	%
Best Practice for all Daily Practice Questions‡	6	24·0	2	8·7	8	16·7
Provider never takes child away from physical activity ≥5 min to manage behaviour†	20	76·9	14	60·9	34	69·4
Provider supervises, verbally encourages and often joins in active play to increase activity†	16	64·0	12	52·2	28	58·3
Provider uses physical activity during transitions, routines and activities at all opportunities†	12	46·2	8	34·8	20	40·8
Physical Activity Education and Professional Development	Median	IQR	Median	IQR	Median	IQR
Average Physical Activity Education and Professional Development subsection score‖	3·3	2·5, 3·7	3·2	2·3, 3·5	3·2	2·3, 3·5
	Count	%	Count	%	Count	%
Best Practice for all Education and Professional Development Questions‡	1	4·0	2	9·1	3	6·4
Provider leads a planned lesson to develop motor skills ≥1/week‡	20	76·9	19	82·6	39	79·6
Provider talks informally about importance of physical activity at every opportunity†	6	23·1	6	27·3	12	25·0
Provider completes professional development 2/year on children’s physical activity†	9	36·0	8	34·8	17	35·4
Provider has covered 4–5 topics on children’s physical activity in professional development†	13	50·0	12	52·2	25	51·0
Provider offers families information on children’s physical activity ≥2 times/year†	14	53·9	7	30·4	21	42·9
Provider offers families information on 4–5 child physical activity topics†	9	34·6	7	31·8	16	33·3
Physical Activity Policy	Median	IQR	Median	IQR	Median	IQR
Average Physical Activity Policy subsection score‖	2·5	2·0, 3·0	3·0	1·0, 3·0	3·0	2·0, 3·0
	Count	%	Count	%	Count	%
Best Practice on Program Physical Activity Policy Includes 6–8 Nutrition Topics‡	2	8·3	4	17·4	6	12·8

† *χ*^2^.

‡ Fisher’s exact test.

§ *t*-test (equal variances).

‖ Wilcoxon.

Statistical significance was examined at alpha < 0·05 (*) and < 0·01 (**), to account for multiple analyses.

IQR is reported as 25th and 75th percentiles.

The median self-efficacy score was 16·0 out of 18; a higher score indicates higher self-efficacy (Table 3). Providers with additional staff (56 %) were more likely to report self-efficacy in serving meals family style than were those without additional staff (17·4 %; *P* < 0·01). However, serving meals family style had the lowest self-efficacy (37·5 % overall) across all eighteen items. The majority of providers (85·7–95·9 %) were confident they could provide praise, keep the TV off during meals and provide aspirational best practice nutrition, with the exception of serving vegetables ≥2/d. Areas with lower, yet still quite strong, self-efficacy were serving vegetables 2≥ times/d (77·1 %), letting children decide how much to eat (62·5 %) and leading a planned nutrition lesson (75·5 %).

**Table 3:** Nutrition confidence and barriers of Family Child Care Homes (FCCH) providers in and around Oklahoma City (*n* 49)

	Providers with other staff (*n* 26)		Providers without other staff (*n* 23)		Overall (*n* 49)	
	Median	IQR	Median	IQR	Median	IQR
Confidence score (range 0–18, higher indicates higher confidence)‖	16·5	14·0, 17·0	16·0	14·0, 17·0	16·0	14·0, 17·0
Confidence: sure or very sure responses	Count	Frequency (%)	Count	Frequency (%)	Count	Frequency (%)
Beverages						
Get children to drink more water‡	22	84·6	21	91·3	43	87·8
Limit juice‡	23	88·5	22	95·7	45	91·8
Serve only 1 % milk‡	24	92·3	23	100·0	47	95·9
Serve only unflavoured milk‡	24	92·3	22	95·7	46	93·9
Avoid sugary drinks‡	26	100·0	21	91·3	47	95·9
Foods served						
Serve fruit ≥2/d‡	24	96·0	23	100·0	47	97·9
Serve vegetables ≥2/d†	21	84·0	16	69·6	37	77·1
Serve fried/pre-fried foods 1/week‡	24	96·0	22	95·7	46	95·8
Serve high-fat meats ≤1/week‡	22	88·0	23	100·0	45	93·8
Serve high-fibre foods ≥2/d‡	21	80·8	21	91·3	42	85·7
Serve high-salt foods ≤1/week‡	24	92·3	23	100·0	47	95·9
Serve high-fat or high-sugar foods ≤1/week‡	22	88·0	22	95·7	44	91·7
Meal environment						
Serve meals family style**,†	14	56·0	4	17·4	18	37·5
Can let children decide for themselves how much food to eat†	17	68·0	13	56·5	30	62·5
Always praise or encourage children for trying new foods‡	25	96·2	22	95·7	47	95·9
Give families information on child nutrition and physical activity on varied topics‡	23	88·5	16	69·6	39	79·6
Never watch TV during snacks and meals‡	26	100·0	21	91·3	47	95·9
Nutrition education						
Lead a planned nutrition lesson ≥1/week†	19	73·1	18	78·3	37	75·5
	Mean	sd	Mean	sd	Mean	sd
Barriers score (range 20–60, lower score indicates lower barriers)§	33·2	5·5	34·7	5·0	33·9	5·3
Barriers: Agree a little or a lot responses	Count	Frequency (%)	Count	Frequency (%)	Count	Frequency (%)
Beverages						
If only served water, children would drink enough‡	22	84·6	21	91·3	43	87·8
If juice were limited, children would get enough vitamins‡	25	96·2	20	86·9	45	91·8
Children like low-fat milk‡	21	80·8	16	69·6	37	75·5
Foods served						
Have enough time to make healthy food‡	21	80·8	16	69·6	37	75·5
Fresh produce goes bad too quickly to be served‡	11	42·3	7	30·4	18	36·7
Fresh produce is too expensive‡	11	42·3	9	39·1	20	40·8
Concerned with waste because children will not eat healthy foods‡	10	38·5	13	56·5	23	46·9
Hard to serve healthy foods because children are picky‡	12	46·2	12	52·2	24	49·0
Some dishes taste as good when made with whole grains‡	21	84·0	17	73·9	38	79·2
Children eat unhealthy foods at home, so it is hard to get them to eat health in FCCH‡	20	76·9	15	65·2	26	54·2
Meal environment						
Have enough time to sit at table with children during snack and meal time‡	14	56·0	12	52·2	26	54·2
Children will make too much of a mess if they serve themselves†	13	50·0	15	65·2	28	57·1
Children will waste too much food if they serve themselves‡	15	57·7	13	56·5	28	57·1
Serving food at meal and snacks is the adult’s responsibility‡	14	56·0	14	63·6	28	59·6
Children will decide the right amount if they get to decide how much to eat†	9	34·6	4	17·4	13	26·5
Like the taste of the healthy food the children are supposed to eat‡	20	76·9	19	86·4	39	81·3
Know how to encourage children to try new foods‡	25	96·2	20	90·9	45	93·8
Know how to talk to children about healthy eating‡	23	88·5	19	86·4	42	87·5
Nutrition education						
Have enough time for nutrition lessons‡	18	69·2	20	86·9	38	77·6
Know how to find materials to use to teach children about nutrition‡	20	76·9	14	60·9	34	69·4

† *χ*^2^.

‡ Fisher’s exact test.

§ *t*-test for two independent means (equal variances).

‖ Wilcoxon rank sum test.

Statistical significance was examined at alpha < 0·05 (*) and < 0·01 (**), to account for multiple analyses.

IQR is reported as 25th and 75th percentiles.

Considering barriers, the mean barriers score was 33·9 ± 5·3 (possible range of 20 to 60), with a lower score indicating fewer barriers (Table 3). Many providers reported barriers within food served and the meal environment, but many were able to provide healthy beverage options and nutrition education. The areas with the fewest barriers included knowing how to talk to children about healthy foods (12·5 %), service of water (12·2 %), concern with ability to limit juice (8·2 %) and knowing how to encourage children to try new foods (6·2 %). The areas with the largest barriers included children deciding the right amount to eat (73·5 %), concerns with food waste because children will not eat healthy foods (53·1 %), picky eaters do not like healthy foods (51·0 %), fresh produce spoils too quickly (63·3 %) and fresh produce is too expensive (59·2 %). There were no differences in barriers between FCCH with and without additional staff.

The majority of providers (79·6 %) were ‘confident’ (40·8 %) or ‘really confident’ (38·8 %); they could meet food programme best practices. Only one provider (2·0 %) was ‘not confident,’ while the remainder were ‘kind of confident’ (18·4 %), indicating that providers were confident in their ability to meet food programme best practices. However, the median food programme best practice knowledge score was 9·0 (IQR: 7·0, 10·0) out of 13, and the median percent accuracy was 69 %, which is lower than desired.

As for aim two, there were no statistically significant differences between providers with and without additional staff for nutrition and physical activity practices, nutrition self-efficacy and barriers or food programme knowledge (data not shown, *P* > 0·01).

## Discussion

The current study’s purpose was to characterise nutrition and physical activity best practices along with nutrition self-efficacy, barriers and food programme knowledge of FCCH providers and explore if there were differences with and without additional staff. In the current study, few met all nutrition or physical activity best practices, although the average scores for nutrition and physical activity ranged from 3·0 to 3·2 (out of 4). These results align with previous reports using self-reported^(11,24,31)^ and direct observation^(15,20,26,32)^ methodologies, confirming the importance of working within this setting to understand the role of the nutrition environment and develop efforts to enhance FCCH nutrition and physical activity support. Many providers were confident in serving healthy beverages and providing nutrition education to children. Providers reported more barriers to food served and meal environment, which support previous literature noting that childcare providers, and FCCH specifically, report barriers and difficulty in creating supportive mealtime environments and practices^(10,13)^. Providers reported insufficient knowledge of the food programme best practices, but indicated they were confident in their ability to administer the food programme best practices^(10)^. There was one difference between providers with and without additional staff, in self-efficacy around family-style meal service. Though contextual differences may exist between FCCH with and without additional staff, all providers may benefit from additional support to achieve nutrition and physical activity practices within this setting.

FCCH providers reported that they strive to serve children healthy foods, consistent with previous work^(13,33)^. Most providers indicated that meal preparation occurred while providing care for children, emphasising the necessity of efficient and multi-tasking meal preparation. Previous studies report that 50–67 % of FCCH providers sit and eat with children^(14,34)^, compared with only 7–13 % of our providers who report ‘always’ sitting and eating with children. Jiang *et al*.^(13)^ indicate that FCCH providers believe they should sit with children at meals and eat the same food, although Hispanic providers felt this more strongly. Only 2 % of providers in the current study report Hispanic ethnicity, which perhaps addresses this disparity between the strong belief of sitting and eating with children and the low participation in this behaviour. Further, Jiang *et al.*^(13)^ do not report how many providers actually did sit and eat the same foods, only that they felt they should.

The mean nutrition practices score is similar to that presented in Dev *et al.*^(24)^ in FCCH, although the current sample reported more barriers than were reported in 970 FCCH providers in Nebraska^(34)^. In the current study, 28·3 % of FCCH exceeded the recommendation for juice of no more than two servings/week, which is lower than previous observation and self-reports of 41–67 % of FCCH serving excessive juice^(31)^. Additionally, 91·8 % of FCCH in the current study agreed that if juice is limited, children will still get enough vitamins. This finding is in contrast with Jiang *et al*.^(13)^ reporting that FCCH believe that if juice is limited, children will consume insufficient vitamins. Providers in our study reported less nutrition and physical activity professional development than did providers in previous studies^(11,35,36)^. FCCH that engage in professional development for nutrition and physical activity have environments that are more supportive of healthy eating and movement, emphasising the importance of training focused on the unique needs of FCCH providers^(11,37)^.

Within nutrition best practices, there was a disconnect between meeting aspirational nutrition best practices and their self-efficacy to meet food programme guidelines and best practices. Indeed, many providers reported low adherence to nutrition best practices (36·7–53·1 %), specifically for serving fruits, vegetables and whole grains, whereas they reported high self-efficacy (77·1–97·9 %) to serve these foods. Providers did report higher adherence to aspirational best practices of limiting unhealthy foods (71·7–85·7 %) and equally high self-efficacy (91·7–95·8 %) to do so. Williams *et al.*^(10)^ reported that there was no difference in meeting self-reported food programme best practices between those programmes participating in the food programme or not. This disconnect may stem from a discrepancy between required training focused on food programme compliance and training addressing optimal nutrition and best practices. Furthermore, technical assistance to implement best practices for foods served and nutrition environment in FCCH is not broadly available.

Providers reported high self-efficacy in implementing the food programme best practices. However, their actual knowledge of those best practices was rather low (69 %). Other literature has similarly described the discordance attitudes and beliefs around children nutrition and actual provider practices^(4)^. The food programme knowledge was lower than anticipated, as all providers are required to participate in annual food programme training. While not directly evaluating the same constructs, few met the aspirational education and professional development nutrition best practices. Participation in the food programme is associated with children’s enhanced nutrition^(10)^ and best practices^(10,11)^ as measured by self-report. Therefore, the current study adds to the literature that FCCH food programme best practice knowledge can be improved, and intervention and technical assistance to enhance best practice implementation may contribute to enhanced nutrition environment.

The context of nutrition and physical activity within these settings may provide insight into these lower scores. Though FCCH providers with and without additional staff differ in the number of children served, they reported similar meal preparation techniques and activities to engage children during meal preparation. To occupy children during meal preparation, most providers reported allowing free-play, followed by watching TV. However, the ability to allow free play may depend on existing equipment; only 36·7 % of providers reported always having portable toys available. TV use during meal preparation and mealtime ranged from 25 to 53 % in our study, which is substantially higher than other studies^(24,26,31)^. This may be due to engaging in fewer professional development activities, as in our sample only 35·4 % of providers complete professional development on children’s physical activity two times annually. Transition time between activities is often sedentary; thus, building physical activity into transitions is a viable way in which to increase overall daily movement^(38)^. Using this time to promote physical activity could reduce time spent in sedentary behaviour, such as sitting and watching TV, further improving providers’ time spent in physical activity and sedentary behaviour best practices. Even reductions in 10 min of time spent sitting with moderate to vigorous physical activity are related to better health in this age range^(39)^. Providers may believe that children are naturally active on their own and do not need additional support or encouragement, as found in another Oklahoma sample of childcare centres^(40)^.

There were few differences between providers with and without additional staff regarding nutrition and physical activity practices. This finding is unexpected, as it seems the addition of another staff member would alter these environments, either positively or negatively. However, FCCH with additional staff are also serving more children, which may negate any benefit from additional staff. Notably, there was a disparity between FCCH with and without additional staff in a hallmark of a family meal environment, provider self-efficacy in allowing children to feed themselves (56·0 % and 17·4 % for providers with and without additional staff). This difference indicates that having additional staff permits greater provider self-efficacy in allowing children greater meal autonomy, which is essential in meeting best practices of family-style meal service. An additional staff member may provide support to supervise young children feeding themselves (e.g. pouring drinks and selecting food), though still only half of those with additional staff members felt confident in this area. Serving oneself is seen to provide benefit to fine motor skills from handling utensils, but also self-regulation skills to determine the amount needed^(41)^. Considering the importance of fundamental motor skills and self-regulation within this age range, supporting this continued skill could have long-term implications. Opportunities to support both FCCH providers with and without additional staff to facilitate this important skill is clearly needed within this environment.

A brief discussion of study strengths and limitations is warranted. Few studies have examined the FCCH environment and provider practices due to accessibility and recruitment difficulty^(42)^. FCCH offer a unique context distinctly different from centre-based programmes. The focus of the current study on FCCH providers is a strength and builds on the nascent body of literature in this environment. The current study also assessed a critical component of the FCCH environment, food programme knowledge, self-efficacy and barriers. Further, the current study included physical activity practices to thoroughly address child energy balance within these settings. Limitations include the cross-sectional nature of the study design, relatively small sample size of FCCH, use of self-reported measures and location in one Midwestern metropolitan area. These constraints limit evaluation of causality and generalisability and possibly limit observance of differences between groups (i.e. with and without additional staff). The sample may be subject to selection bias, where those who chose to participate may have healthier practices. Recruitment materials sought FCCH providers interested in enrolling in an intervention to improve either environmental health or nutrition environment with random treatment assignment. No data on the demographic characteristics of FCCH who did not volunteer are available for comparison. Reliance on self-reported measures is subject to social-desirability bias and thus may overestimate best practice achievement^(43)^. No validated instruments to evaluate the food programme knowledge or FCCH provider nutrition self-efficacy or barriers were available; however, internal consistency of the scale was acceptable (0·70–0·84). Even so, the nutrition and physical activities practices and policies tools are psychometrically strong and have been used in other studies^(13,28)^. FCCH provider food security status, use of public assistance programmes and personal preferences regarding nutrition and physical activity were not collected and are outside the scope of the current study.

Several practical implications and scientific research questions have emerged from the current study. One implication is the opportunity to support children’s physical activity within these transition periods of meal preparation. Opportunities to support physical activity and reduce time spent sitting could improve FCCH best practices. These changes also align with recent international guidelines on the 24-h movement cycle in young children^(44)^ and recent changes to other state policies to support less screen time^(45)^. Adding clear movement and screen-time guidelines to the state licensure requirements is a likely opportunity to enhance FCCH quality^(46)^. A second implication is supporting FCCH in foods served and their meal environment, including methods for FCCH providers to support children serving themselves, specifically addressing barriers to children serving themselves, such as the perceptions of mess and food waste. This may be a large transition for FCCH providers, especially those without additional staff, and nutrition education professionals are encouraged to consider the context and gradual transition to support FCCH providers in adopting these changes for long-term success. Finally, the lack of awareness and knowledge of the CACFP, especially regarding best practices, is a clear area of improvement. This deficit may be due to training and education being designed for centre-based care. Despite 25 % of children receiving care in FCCH^(47)^, many training and education programmes are designed for centre-based programmes. Actual food programme best practice knowledge was lower than anticipated, indicating the opportunity for enhanced learning opportunities that are tailored to the FCCH environment. Understanding how food programme self-efficacy and knowledge impact meeting food programme requirements and best practices would be an important next step in this research. An important area for future research is the role of FCCH providers’ food security, use of public assistance and personal dietary and physical activity habits and preferences in the inclusion of nutrition and physical activity best practices in their FCCH. Childcare teachers have a high prevalence of food insecurity^(48)^ and poor nutrition and physical activity habits^(49)^ and report that they often struggle with nutrition^(50)^ and interest in physical activity^(21)^. While these studies have examined childcare teachers in general, they have not examined FCCH providers specifically, nor the impact on their FCCH environment or quality of care. A final comment regarding implications of these findings is in regard to the contextual state environment of Oklahoma and the USA. Oklahoma is a rural and suburban state. Even in the metropolitan areas, there is often ample outdoor space, and licensing requirements align with the old food programme meal pattern^(5)^. Additionally, in the USA, ECE programmes have the support of the food programme. Consideration of how FCCH in lower income and developing countries may include best practices for nutrition and physical activity and FCCH self-efficacy and barriers warrant future exploration.

## Conclusion

Few FCCH providers, with or without additional staff, met all nutrition and physical activity best practices. Providers had high self-efficacy in providing optimal nutrition and engaging in healthy feeding practices and education, although some barriers were still present. Opportunities and resources for FCCH providers to meet food programme best practices and aspirational best practices are warranted to help enhance the FCCH environment. Food programme knowledge was lower than anticipated, given that participation in the food programme was an inclusion criterion. Detailed understanding of nutrition and physical activity environment predictors, including context and staff availability, and association with child outcomes are necessary future directions. Further, interventions to enhance the health and quality of FCCH environments targeting the unique considerations of the FCCH need to be developed and evaluated. Interventions developed should consider implementation sustainability and ability to scale, as well as integration within existing childcare support infrastructure, such as resource and referral professionals and events and training opportunities coordinated by food programme sponsoring organisations. Providers may benefit from future training and continuing education developed with stronger interprofessional integration of both education and nutrition professionals to enhance FCCH health environments.

## Supplementary material

For supplementary material accompanying this paper visit https://doi.org/10.1017/S1368980022000337. click here to view supplementary material
